# The Unmet Needs of Community-Dwelling Stroke Survivors: A Systematic Review of Qualitative Studies

**DOI:** 10.3390/ijerph18042140

**Published:** 2021-02-22

**Authors:** Yunfei Guo, Zhenxiang Zhang, Beilei Lin, Yongxia Mei, Qingxuan Liu, Leyun Zhang, Wenna Wang, Yuan Li, Zhongrong Fu

**Affiliations:** School of Nursing and Health, Zhengzhou University, Zhengzhou 450001, China; togyfei@163.com (Y.G.); linbeilei.com.cn@163.com (B.L.); myx@zzu.edu.cn (Y.M.); liuqingxuan96@126.com (Q.L.); 15937197580@163.com (L.Z.); 202011401030449@gs.zzu.edu.cn (W.W.); a1321270190@126.com (Y.L.); la97@gs.zzu.edu.cn (Z.F.)

**Keywords:** stroke, unmet needs, community health, qualitative research, systematic review

## Abstract

The unmet needs perceived by community-dwelling stroke survivors may truly reflect the needs of patients, which is crucial for pleasant emotional experiences and a better quality of life for community-dwelling survivors not living in institutionalized organizations. The purpose of the study is to identify the scope of unmet needs from the perspectives of stroke patients in the community. A qualitative meta-synthesis was performed according to the Joanna Briggs Institute method. Six electronic databases were searched from inception to February 2020. A total of 24 articles were involved, providing data on 378 stroke survivors. Eight categories were derived from 63 findings, and then summarized into four synthesized findings based on the framework of ICF: (1) unmet needs regarding with the disease-related information; (2) unmet physical recovery and activity/participation needs; (3) unmet needs for social environmental resources; (4) unmet psycho-emotional support needs. We found the framework of ICF mostly complete, but unmet information needs still remain. The needs that are mainly unsatisfied include physical, psychosocial and informational, as well as the practical support from professional or environment resources. The ever-present unmet needs perceived by community-dwelling stroke survivors who do not live in institutions are discoverable and mitigable. Future studies should focus on quantifying unmet needs comprehensively derived from experiential domains, assessing the rationality of the unmet needs expressed by patients’ perspectives and developing flexible strategies for long-term and changing needs.

## 1. Introduction

As the second leading cause of death worldwide, stroke is also the leading cause of disability, and it has produced numerous disasters worldwide [1,2]. Based on the Global Stroke Statistics [3,4,5], the global crude number of novel stroke events has suggested a year-on-year increase, especially in low and middle-income countries. Meantime, though medical technology and health service have made great progress, it has always been the common choice for stroke survivors to discharge the community after the hospital stay [6,7,8]. However, there are some inevitable problems when they come home. They still face a long and tedious recovery process (e.g., the return of physical, speech, cognitive other functions) [9]. They still have several unmet needs that should be met, as described in the study on discharge preparation [10] or transition care [11].

Existing studies explained unmet needs as a need of something or help from someone (which would help overcome some of the effects of stroke and resulting difficulties) that is not being met [12,13]. Numerous studies [14,15,16,17] delved into the unmet needs’ contents of stroke patients (e.g., unavailable preventive services, professional support and invention, emotional and information communion, as well as level of unmet needs). It is highly sophisticated and multifactorial. Unmet needs recognized by nurses, physicians, or patients are significantly different. Claude Vincent [18] interviewed four populations, i.e., older stroke people, caregivers, health professionals, and health care managers, highlighting the four populations reported with their special perspective for the unmet need of stroke patients. For instance, Abrahamson V [15] reported that stroke nurse specialists emphasized investigations, medication, and liaising with general practitioners or consultants, while the stroke association coordinator highlighted signposting to other services. Of note, some researchers [15,19] reported that clinicians may sometimes discover unmet needs; however, if the patient believes that further intervention will not alleviate the need, they will not find it, which means that the patient’s unmet need is irreplaceable and unique.

Unmet needs have not been systematically examined in relation to stroke patients in the community. Existing syntheses of qualitative studies in stroke have focused either on the patient experience of rehabilitation, instead of unmet needs [20,21], or on the experience of care [22,23,24]. Few syntheses systematically reviewed the unmet need of stroke patients [25,26] (e.g., the identification of needs in the acute phase, rehabilitation phase, or long-term unmet needs), whereas they did not state the unmet needs in the community. To the best of our knowledge, no studies have summarized the literature on the perceived experiences of unmet needs of community-dwelling stroke people who do not live in institutions. This study focuses on the unmet needs of patients’ perspectives in the community and adds the psycho-emotional needs. It is of great interest to conduct an unmet needs assessment, which is to enhance patient outcomes for body functioning, activities, participation, and quality of life. Thus, this systematic review aimed to identify and synthesize the qualitative study of the patient experience of unmet needs in the community.

## 2. Methods

### 2.1. Protocol and Registration

The initial protocol and systematic review were prospectively registered, available at an existing study protocol [27] (Record: CRD42018112181) This systematic review was conformed to the JBI Critical Appraisal Checklist for Systematic Reviews and Research Syntheses [28] (Supplementary Table S1) and reported in accordance with it.

### 2.2. Search Strategy

The review methods have been described in detail elsewhere [27]. Searches were conducted with the following electronic databases: PubMed, EMBASE, CINAHL, PsycINFO, SCOPUS, and CBM (China Biology Medicine) from inception until February 2020. The English and Chinese language restriction were due to a lack of funding for translation. We focused on three key concepts (stroke, needs, community) in accordance with published protocol; we adapted our search strategy for each database, and the final statement was in the following form:

- Stroke (cva* or stroke* or poststroke* or post-stroke* or post stroke* or transient ischemic attack* or TIA* or ministroke* or mini stroke* or SAH or cerebral or cerebellar or brain* or vertebrobasilar or infarct* or ischemi* or thrombo* or apoplexy or emboli*);
- Needs (need* or demand* or requirement* or wish* or experience or challenge*);
- Community (community or home or outpatient*).

The reference lists of all included articles and relevant systematic reviews were scrutinized to identify additional studies for potential inclusion in this systematic review.

### 2.3. Selection Criteria

Criteria to select material for inclusion in the review included: (1) studies on unmet needs that investigated directly from stroke survivors or partly from them; (2) any qualitative and mixed-method research method used (e.g., interview, focus group, ethnographic); (3) published in a peer-reviewed journal and written in the English or Chinese language; (4) participants are human, with a diagnosis of stroke; (5) individuals aged over 18 years; (6) study participants are living in community-dwelling or at home; and (7) in these studies, some patients who lived in institutions and others will be included only if it is possible to extract data separately and without mingling with caregivers or professionals.

**Figure 1:**
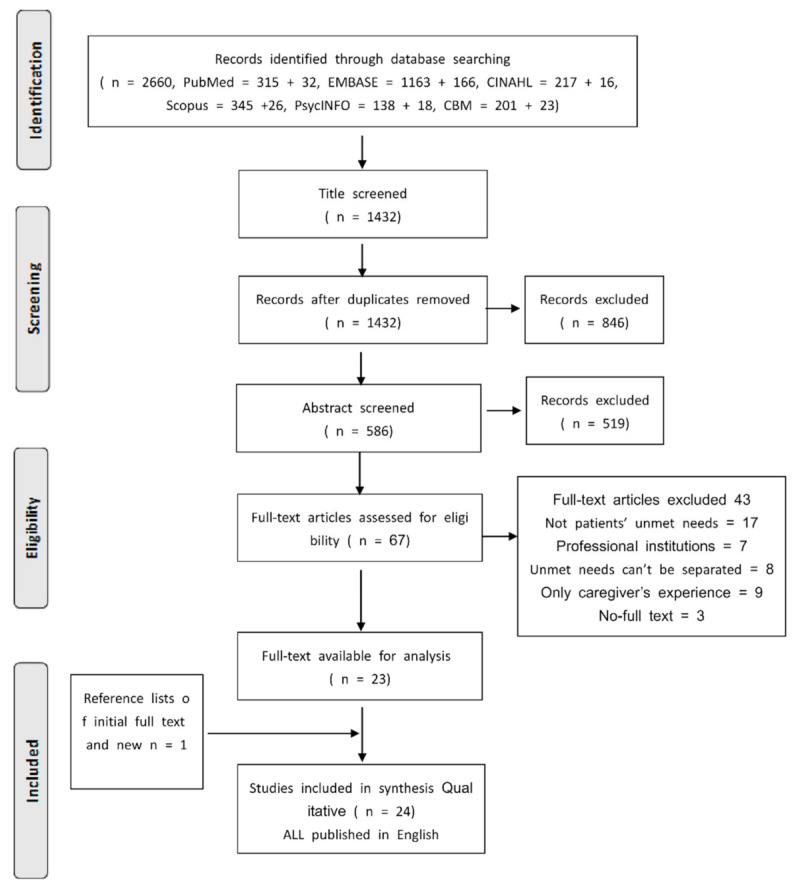
Literature identification process.

Criteria to select material for exclusion in the review included: (1) published abstracts excluded and (2) all participants living in any institutionalized organizations. (see Figure 1).

### 2.4. Selection of Articles for Inclusion

After the removal of duplicates by main reviewer, all titles and abstracts were scrutinized independently by two authors (Y.G., B.L.). If there was insufficient information to determine ineligibility of an article or if the information from the abstract suggested that it might be eligible, the full article would be obtained. In the next phase, full-text articles were screened independently by two authors, and a third reviewer would join in to read all divergent articles and a consensus was conducted among reviewers if differences emerged (Y.G., B.L., Q.L.)

### 2.5. Data Extraction

Two authors built a matrix to record the following data for each reviewed paper (e.g., publication year, countries, sample characteristics, study methodology, data collection, research content and the details of unmet care needs). If a study containing data from two or more populations (patients, caregivers, health professionals, or health care managers), we only extracted the data of patients when the unmet needs came from the perspectives of different populations. Lastly, the two-table file was compared by another review. Differences in data extraction between authors were addressed by discussion or by a third author.

### 2.6. Data Synthesis

Details of this thematic synthesis were reported in an existing study protocol [27]. In brief, the data synthesis process followed three critical stages (i.e., data coding, development of descriptive themes, and generation of analytical themes) [29,30]. A thematic analysis developed with an inductive approach was employed, which was independently analyzed by two or more researchers (Y.G., B.L., Q.L.). Qualitative data were thematically analyzed with the qualitative meta-synthesis method capable of systematically reintegrating the primary findings. Specific codes or themes for unmet needs domains were synthesized. Unmet needs reported were categorized under four meta-themes (i.e., information needs, physical recovery/participatory needs, environmental resource needs, and emotional needs), which partially complied with the International Classification of Functioning, Disability and Health (ICF) Core Sets for Stroke framework [31]. Two reviewers would discuss the mentioned items; besides, if they cannot reach a consensus, a group discussion should be raised.

### 2.7. Assessment Quality of Involved Studies

The quality appraisal according to the Joanna Briggs Institute Qualitative Assessment and Review Instrument (JBI-QARI) is based upon published guidance by the well-known institution [28]. This instrument comprises a checklist devised to identify the possibility of bias in its design, conduct, and analysis [32]. The same two authors (Y.G., B.L.) independently assessed the involved articles for comprehensive reporting. Disagreements between the two reviewers were addressed by discussion or by resorting to the judgment of a third reviewer if needed (Q.L.).

## 3. Results

### 3.1. Characteristics of Included Study

In total, 2660 original records were identified. After duplicates and any articles not published in journal format, 1432 titles remained. To be specific, 67 articles were retrieved as full-text articles, and 24 subsequently met our inclusion criteria. (Table 1). The Prisma flowchart (Figure 1) outlines the article selection process. Of the 24 studies involved, 5 were conducted in in the UK, 4 each in Australia and Sweden, 3 each in the USA and Canada, 2 in Norway, and 1 each in Malaysia, Italy, and Iceland. Most (10) of the studies employed semi-structured interviews, 5 used in-depth interviews, 4 applied focus groups, 4 adopted mixed-methods, and each survey was conducted by raising an open-ended question. Note that studies were published between 1991 and 2019 with most (12/24) published after 2015.

The number of participants ranged from 4 to 46 participants (with overall 378 participants recruited), and most (18/24) had participants fewer than 20. Across all studies, the age of all participants ranged from 19 to 95, all the patients ranged from 0.5 months to 24 years after stroke or discharging home. Among all published journal articles identified finally, 9 studies covered mixed populations, most (6) of which combined patients and their caregivers [33,34,35,36,37,38]. Two studies consisted of four populations [15,18] (i.e., patients, caregivers, health professionals, and health care managers), 1 covered three types of participants [39] (i.e., nurses, physicians, and occupational therapies), 1 contained patients and professionals [15]. Importantly, we only focused on the data of stroke patients. Besides, 6 studies had participants with a special population [35,40,41,42,43,44] (e.g., aphasia, cease driving, and cognitive and emotional symptoms). Two studies were longitudinal qualitative interviews [36,45]. The present study characteristics and synthesized findings were summarized specifically in Table 1.

### 3.2. Meta-Synthesis

**Figure 2:**
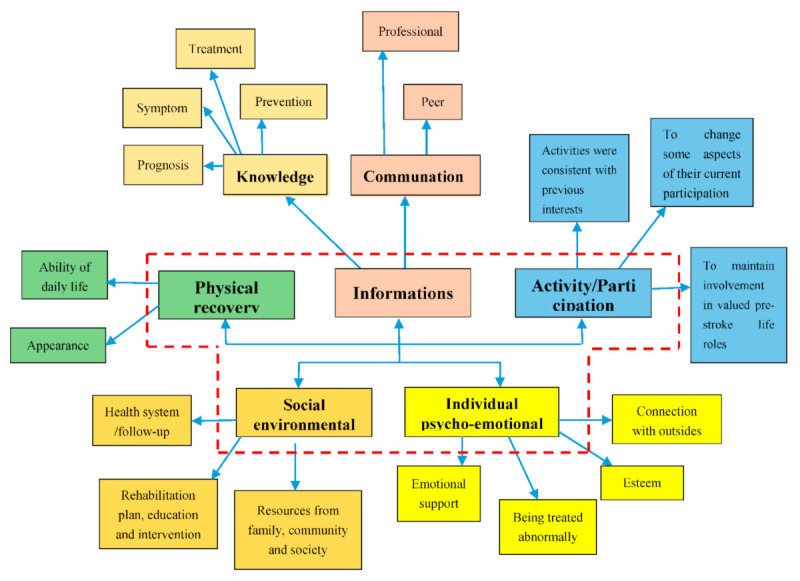
Coding tree.

All the extracted findings were classified as yes, no, not applicable or unclear regarding the quality of evidence (see Table 2). From the 24 quantitative studies that assessed unmet needs across multiple domains, these findings were aggregated into 8 categories that were combined in the following four synthesized findings: (1) Stroke people at home have unmet needs about the disease-related information; (2) stroke people have unmet participation/physical recovery needs; (3) stroke people have unmet needs of social-environmental resources; (4) stroke people have unmet emotions needs. The categories and synthesized findings are listed in Figure 2.

#### 3.2.1. Stroke People in Community Have Unmet Needs for Disease-Related Information

Stroke people referred to not having received sufficient information concerned with disease symptoms, treatment, and prognosis. They required further communication with professionals and peer to guide their decision-making and clear self-situation. Moreover, they mentioned not having received information on some advanced activity/IADL (Instrument Ability of Daily Life).

##### Unmet Needs around Knowledge of Stroke Symptom, Treatment, Prevention, and Prognosis

The participants’ unmet need for stroke-related information seemed insatiable, they generated inexhaustible interest in it (e.g., stroke symptom, treatment, prevention and prognosis), and they did [19,43,45,47,49]. Patients usually want to know many faces and forms of stroke [47], which was due to being afraid of having another stroke or of possible medication side effects [49]. Even more, some patients did not meet the passive mode of receiving information; they wanted more information regarding their illness and treatment to make their own decisions [19]. One patient mentioned that, “I don’t know why the doctor gave me so much medicine... Once you have a stroke, you need to read a lot to know more about it…we don’t know when we can get it again” [19]. However, they sometimes showed a lack of information regarding how to access health-related services about their care and rehabilitation or what were the available assistive devices for themselves [36,45,52]. “This part is critical, where to go and [whom to contact] and what my rights are…I really do not know which assistive devices I need at home” [45].

##### Unmet Needs Regarding Information on Communication with Professional and Peer

A further information gap showed associations with communication with professionals and peers [35,40,43,49]. Stroke patients at home had difficulties in making thoughtful decisions and receiving reliable information support [40], for instance, information regarding alternative transport options and other lifestyle changes. On the whole, they usually had an unmet information need for early contact with a stroke rehabilitation unit, regular follow-ups, and a rehabilitation plan [36,43]. As one person expressed, “You’ve had very little information regarding, about what to expect… I have problems with my right hand… then I’m very…I get very tired as well. and that’s also–will that go away? There’s no one who can really give an answer… I’ve never gotten any explanation” [43]. Note that patients also had an indispensable need for information from a peer. By talking with others, they ascertained what was likely to happen to them, what difficulties were normal for someone at their ages, and they were enabled to gather and use the information to reflect on their situation [48]. “You see people with arms that don’t work down there in different forms and some can’t speak properly, so I was pleased with that” [48].

#### 3.2.2. Stroke People Have Unmet Physical Recovery and Activity/Participation Needs

Stroke people are referred to having difficulties in participating in various activities. They felt a sense of inadequacy during activities, as they were not able to participate as they did before. Furthermore, they were yearning to become normal, regain pre-stroke ability of daily life and get involved in their valued pre-stroke life role.

##### Unmet Activity/Participation Needs

Stroke people expressed the need to engage in a variety of activities and to continue as much as possible to get involved in all the activities [15,44,49,52]. They tended to engage in activities that were consistent with their previous interests [44]. As one patient expressed, “We must continue to do what we were doing before, activities or anything else, if we are able to” [44]. It is noteworthy that the interesting phenomenon is that stroke patients usually hope to continue engaging in activities they had appreciated before the stroke [44,49]. To some extent, they may not be able to do it as well as before, but they still want to change some aspects of their current involvement, e.g., to increase the frequency of meaningful physical and leisure activities [44], “I would like my gait pattern to become more natural to be able to perform previous activities without assistance…, I’d taken two [computer] courses to develop the skills to participate in new activities” [44]. Moreover, they struggled to maintain involvement in valued pre-stroke life roles in and outside the home [15,52]. “We need to be able to try and get back at it [work] as soon as we can” [15].

##### Unmet Physical Recovery Needs

Physical recovery was reported as the common unmet needs, showing a relationship to their ability of daily life. They were afraid of the loss of balance, climbing stairs, etc. They wanted to be able to manage the transition from the inpatient rehabilitation facility to community and “become their masters again” [18,33]. One patient mentioned, “I should try their gym department, and I’ve been going there for five weeks now, I’m going there this afternoon” [34]. Moreover, stroke patients had an appearance requirement; they wanted to maintain a good outward façade in front of unfamiliar people [41,53], especially for stroke people subjected to eating difficulties, “if I’m elsewhere I’m extremely conscious that there is no food dripping. I don’t realize if I have tomato sauce all over the place. I don’t sense it. I prefer to eat [pause] not like a child” [41].

#### 3.2.3. Stroke People Have Unmet Needs for Social Environmental Resources

##### Unmet Health System/Follow-up Needs from Professionals

Stroke people reported that they had difficulties in accessing dedicated healthcare services; there existed a gap of advice, supervision, and intervention from the health system to meet the unpredictable and persisting needs [15,18,34,35,36,37,38,40,42,44,51,54]. A lack of difficulty in accessing the services and obtaining follow-up from the health system when patients at home were commonly mentioned by most patients [18,35,37,44]. They needed home care assistance and supervision from the health system to avoid the feeling of abandonment; they desired to get a backup: “It’s a long time to wait before they came round, I wanted to get moving because the physio was so good in hospital … but then when you come home there’s nothing…I wanted to just get going and build on what I was doing in the hospital” [15]. However, even if the resources were available, sometimes individuals reported unmet with the contents of services or recourse they had received. They required a long-term, continuous, and practical follow-up [34,44] as described, “I was very sorry that it stopped because I felt that there was no more communication, nobody would see how I was getting on from that point other than let’s say, my local doctor” [34]. Furthermore, patients might have some personalized needs; [39,41] for instance, stroke patients with aphasia raised an unmet need for a flexible follow-up system which could suit their family life situation and disease severity [51], as an attempt to pursue some tailored services, “I can’t tell her, that she should not [go to work] because I’m going to my follow-up. So [the follow-up system] has to be flexible, if it’s going to work” [51].

##### Unmet Family, Community, and Social Needs for Environment Resources

Settling down in community, some stroke patients reported that they desired to gain a suitable living environment [33,50]. They required security; they hoped their homes could be changed to adapt to their disability [50], and even the care environments could adopt their original culture [38], “Traditional Chinese medicine to be more slow acting than conventional methods, but efficient…A Chinese diet that was balanced in ‘yin’ and ‘yang’ foods would keep the body in balance, and that a misbalanced diet (e.g., hospital diet) could give rise to illness” [38]. Furthermore, individuals reported limited access to transportation; the seats on trains and buses were full; disabled parking spots were commonly taken, especially for those who had long walking distances to public transportation [40,43]. “I wasn’t allowed to drive a car, which immediately led to long days when you had to take the bus and it was a long walk to the bus. I thought that was the worst part, getting to and from work” [43].

##### Unmet Rehabilitation Plan, Education, and Intervention Needs

Specific to stroke people, the gap of early information regarding stroke and its consequences could create the flowing of needs [43]. As they suggested, a rehabilitation plan at an early stage after stroke onset would facilitate return to work (RTW), “It would have been good to get a plan earlier. To receive [a rehabilitation] contact earlier” [43]. Besides, they also desired to acquire educational services whatever physical training or disease-related knowledge [15,38,44]. For instance, they should improve their specific abilities by learning exercises (e.g., walking, dexterity, and language). As one patient stated, ”I like that [written information about exercises] because it tells me how to do it. With that, I’m okay and they had me do the exercises” [44]. They also reported that they needed to be monitored closely; they strived to receive timely, continuous and personalized interventions [15,44,51,54], “After the kinesiology activities, the aquafitness to rebuild my muscles, I would have liked specific workshops” [44].

#### 3.2.4. Stroke People Have Unmet Individual Psycho-Emotional Support Needs

Stroke people usually had many types of psycho-emotional needs; they reported that physical function limited their social activities and led to greater social isolation; they felt unfreedom, worries, depression, anxiety, emptiness, abandonment, confusion, frustration, and so on [33,37,39,42,46]. For instance, Patients expressed a sense of emptiness after returning home and a feeling of being left behind, “you’re in hospital for a week and then you’re just released into the wild. and then there’s no specialist or stroke staff there to meet you until you get to see the nurse” [39]. They wanted to contact their friends and family; they needed the emotional support of family, friends, or professionals. Furthermore, for concerns about being treated abnormally, individuals desired normality and belonging from others. They considered it uncomfortable, embarrassing, and hard to master eating situations in front of unfamiliar people [53]. As one people stated, “it’s embarrassing if you go out to a meal…yes, I mean, people look at you and wonder…like has she got to have her food cut up” [53]. Interestingly, even some normal behavior can also be considered unreasonable, “’oh can I open the door for you’… it just… reminds you that you’re different” [47]; they just wanted to feel normal again.

## 4. Discussion

This systematic review provided qualitative evidence of the experiences of stroke people regarding their perception of unmet needs. The community-dwelling stroke survivors in this study are special; they represent the group of people who do not live in institutions. Our study identified that the special stroke survivors have a broad range of such needs, covering informational, participation, environmental resource, and emotional needs.

Unmet information needs have been the most frequently mentioned area when stroke patients return to community living [26,38]. It has been suggested that stroke patients have various unmet information needs (e.g., clinical information, practical information, and information on continuing care and resources in the community) [36,51]. Critical information helps improve quality of life, empower patients to feel more in control of their disease and alleviate distress upon returning home concerning how to manage their symptoms [42,55,56]. The lack of communication means participants living with incorrect or limited information; it may arouse ongoing feelings of uncertainty and distress about the management of symptoms following discharge [42]. For the information, it is necessary to face the mentioned problems (e.g., provision of information is lacking, inadequately allocated time, inappropriate timing, or incomprehensible information) [57]. For information transmission, differently delivery styles for information exert a different role; the optimal way to convey information should be taken to achieve the desired results [45,58]. For instance, face-to-face, written, and the internet are prioritized for information concerned with practical management strategies. However, even if the information is accessible, the issue of stroke patients still not been resolved sometimes as they are significantly bad at expressing their needs or they do not really keep the message in heart. They know nothing about the types of information they need for cognitive, insufficient difficulties, and psycho-social issues or low health literacy [39,45,59,60]. It was demonstrated that education, combined with counseling for self-assessment, could help people be more aware of their needs [18]. Patients should acquire unmet information by receiving from professionals and family, as well as by sharing experiences with other patients [61].

With the return to the community, the focus of stroke patients gradually shifted to daily life. Physical rehabilitation needs are the basic needs for stroke patients. They hope to live a life as close as possible to the one they lived before the onset of their disease [44,62,63]. Such as leisure outings (e.g., fishing, golf, driving) or activities of personal pleasure (e.g., reading and listening to music). As a matter of fact, the profile of participation in leisure and the level of unmet needs vary among participants [44]. Thus, it is critical to provide personalized and responsive education about participation in leisure activities to guide them to choose and try new and challenging leisure or sports activities according to their motivations, interests and passions [44]. Moreover, professionals are in a crucial role in guiding the patients’ bodily recovery, making sense of their altered body, and cultivating their confidence [49], and caregivers could build a bridge for patients to access various locations and social networks [40]. In particular, some unmet needs of social participation (e.g., RTW, driving, and eating outside) need a timely and professional assessment to determine whether the patients are fit to participate or stop them from bad experience (embarrassment, stigma, etc.) [40,42,43].

To receive some assistance in adapting to the new situation or avoid being abandoned by the health system, they usually want to make a connection with social-environmental resources [15,51,54]. There are various connections, covering practical education, flexible invention, long-term follow-up, and rehabilitation plan, from health system or professionals [35,43,44,50,51]. This is a huge area of unmet external demand that should be rigorously considered. Suitable living conditions are an elementally environmental element [33] (e.g., support from family members, public service, and family rehabilitation environment) [33,38]. After discharge, some expressed concern regarding service cessation and who would monitor their progress [18,34]. Most importantly, sufficient support should be given to patients and caregivers to identify the resources available to them in the community [18,37].

Moreover, the health system should keep a connection with stroke patients through continuable, personalized, long-term, and practical services, to meet their changing needs [34,39,44,52]. This would ensure that they were indeed assisted and do not feel isolated [51]. Moreover, the invention, rehabilitation plan, and education from professionals or caregivers are also some of the unmet needs [44], and those consist of the main connection with professionals or caregivers. Meantime, health professionals should consider the deficits or recovery after stroke and adapt the education process accordingly [40].

In the present review, unmet emotional needs are highlighted. Affective issues after stroke are also commonly experienced and often endure for many years after the illness event [47]. Patients expressed a sense of emptiness after returning home and a feeling of being left; it was reported that psychosocial problems are more difficult to rehabilitate than physical problems [39,43]. Negative psychological problems are related to terribly physical symptoms, perceived impairment, and so on [42,47], which could lead to greater social isolation or be constrained through declining communication or participation [42]. Early and ongoing access to psychological care is pivotal for patients [39,47], Health care workers believe that a lack of follow-up can lead to an increase in negative emotions. Ongoing symptom monitoring or assessment needs to be integrated into existing community services to address emotional symptoms and provide appropriate interventions to improve emotional self-regulation [46], Moreover, the emotional need could be met by re-mastering their situations [39]. However, patients were sometimes reluctant to seek help for psychological issues, to simplify the process of accessing help and express mood timely [64].

The findings of this qualitative review showed the need to create a practical “connection” that meets the authentic needs of stroke people. The first thing we need to know is that the unmet need is not equal to problems caused by stroke. Because some participants say they have no outstanding needs, others may have the same problem. They also have a different attitude, self-management skills, and support from external resources environment. Though needs will never be met, this may continue to provide increased support if conditions permit. Some of these requirements represent unmet requirements, including those that are reported as unmet or that they are not aware of [65,66]; furthermore, patients, caregivers, and professionals all have unique perspectives in their own opinion. Thus, the unmet needs identified by one group may be incorrect or unreasonable or unconscious. Moreover, the needs’ expression, efficient institution, and consciousness of expressing needs, are critical elements, only in this way can professionals or caregivers receive the unmet needs accurately. At the same time, it is very important to use the qualitative and quantitative methods to conduct a dynamic assessment of the body function as well as the environment, social participation, leisure activities, interests, emotions, and personalized needs to create the most appropriate follow-up, education or intervention to satisfy the unmet needs of patients.

### Strengths and Limitations

Our review has some limitations. Firstly, the data included may be limited since we give up the articles without a clear narrative of unmet needs or articles just displaying problems or issues, because some needs are not the unmet needs, or stroke survivors may still have a persistent need after their unmet needs are met. We considered various needs in this paper. In this way, we could synthesize the most accurate unmet needs. Secondly, the concepts of unmet needs are inconsistent and relevant [26,67], and the country of origin was relatively limited. To be specific, the studies were primarily reported from developed nations, and only one originated from a developing nation. Therefore, it may not be fully generalizable to other nations with different ethnicities, healthcare systems, and payer models. Thirdly, we could not analysis the relative importance of various categories according to the original references, which is regretful. Finally, the present review, by only including four pieces of longitudinal research during the discharging to home phase, made it impossible to monitor changes in unmet needs. However, this is the first systematic review that has identified the perceptions of stroke people of the types and causes of their unmet needs, and there are some valuable findings that quantitative investigations do not illuminate.

## 5. Conclusions

The present review presented an emerging consensus on the unmet needs of stroke people, displayed their experience or the concerns of the public health service or home care resource indirectly, and created a comprehensive taxonomy of unmet needs, which may potentially stimulate service improvement. The present study generated eight categories of unmet needs which contributed to identifying potential unmet needs of community-dwelling survivors who do not live in institutionalized organizations. Integrating the unmet needs is vital to know the scope of their subjective needs, to meet personalized needs, and to improve the community nursing program for stroke patients. The current research on unmet needs still lacks personalized evaluation of patients’ views and specific coping strategies. The unmet needs may be relieved, if the following measures are taken:

(1) Developing a management program for community-dwelling stroke survivors who do not live in institutionalized organizations to build a communication channel for patients and professionals.
(2) It is necessary to provide scientific participation/daily activity/ physical recovery plans for patients with personalized unmet needs, which is the patient’s insurmountable knowledge gap especially for stroke survivors who have no access to professionals.
(3) Regular comprehensive assessment, not only physical needs but psycho-emotional and social environmental resources, plus individual evaluation of unmet needs.
(4) A long-term, continuous health service, which could propose practical suggestions or assistances.
(5) We need to identify short-term needs and long-term needs, that is to say, some unmet needs may disappear after efforts while some unmet needs may reappear soon.
(6) We need to compare the unmet needs from various perspectives (e.g., professional, caregivers, family members). To some extent, the perspectives from patients or caregivers are inconsistent with those of professionals. Then, we may design optimal coping strategies.

The unmet subjective needs of community-dwelling survivors who do not live in institutionalized organizations include a huge scope, and we still have a lot of details to work out.

## Figures and Tables

**Table 1:** Characteristics of studies involved (*n* = 24).

First Author	Country	Population	Methodology	Data Collection	Research Content	Total Unmet Needs
**Valérie Poulin** [44] **2019**	Canada	20 community-dwelling stroke people; range 60–88 years; discharged from rehabilitation units >12 months	Framework analysis	Mixed methods. Semi-structured interviews	Educational needs for support their participation in leisure activities	Tools and resources promoting active involvement, interactions, and opportunities for choice and control; engage in different personally meaningful activities; follow-up to address their educational needs with respect to participation in leisure activities that promote cognitive health.
**Monique R. Pappadis** [46] **2018**	United States	40 community-dwelling older adults at least one-year post stroke; average (43% of the sample was 1–2 years post stroke) 65.1 years	Thematic content analysis	Mixed methods. Semi-structured interviews	Chronic post-stroke cognition and mood symptoms and goals	Unmet need for cognition or mood-related treatment prevailing need for additional speech/language services
**Emma K. Kjo¨rk** [39] **2019**	Sweden	18 patients; range, 3 months to 7 years since stroke	Framework analysis	Focus group	Barriers to access appropriate treatment and support included difficulties in communicating one’s needs and lack of coherent follow-up	Equal access to health care services to satisfy their communicating needs; receiving support for avoiding negative feelings (e.g., emptiness and abandonment); need for comprehensive, planned, and tailored follow-up
**Gunvor GARD** [43] **2019**	Sweden	20 Participants aged 52 (39–62); in 180 days after stroke onset.	Content analysis	Focus group	To explore stroke survivors’ experiences of healthcare-related facilitators and barriers concerning return to work after stroke.	Adequate rehabilitation content and timing facilitated return to work (RTW); lack of early information, regular contact, and a rehabilitation plan were barriers to RTW; insufficient communication between rehabilitation actors hindered RTW; lack of practical help and psychological support the family were barriers to RTW; requesting rehabilitation planning, healthcare information and coordination
**Jamuna Rani Appalasamy** [19] **2019**	Malaysia	10 informants, aged 57(44–78) years, no detail period post-stroke	Inductive thematic analysis	In-depth individual interviews	To determine the fundamental needs and barriers of medication-taking self-efficacy	To gain insight into stroke recurrence and rationalize how the stroke occurred and why they must adhere to preventative medications.
**Abrahamson, Vanessa** [15] **2019**	England	46 patients aged 28–88 years; 24 were followed for 6–12 months, 15 for 12–18 months, and 7 for less than 6 months.	Thematic analysis	Semi-structured interviews	To explore needs identified by patients	More intensity, frequency and duration of therapy; Information concerned with resuming work to work and financial; the need for ongoing support linked with the need for education along the care pathway; be referred to appropriate specialists when symptoms happened
**Jessica Shipley** [47] **2018**	Australia	19 young participants aged 19 to 54 years at diagnosis and ranged from 6 months to 24 years post-stroke.	Rigorous qualitative descriptive analysis	In-depth semi-structured interviews	To examine the personal and social experiences of younger adults after stroke	Desiring normality and belonging; need for increased awareness of the many faces and forms of stroke
**Shannon R.L** [48] **2016**	England	10 participants 78 (70–95) average 11 months after stroke	Thematic analysis	Semi-structured interviews	To identify stroke-survivor unmet needs	Information regarding other stroke people; communication with family doctor and peers
**Nadia Davoody** [45] **2016**	Sweden	4 participants aged 65–85, time from first stroke >10 years	Content analysis	Focus groups	To explore post-discharge stroke patients’ information needs	To have access to health-related information concerned with their care and rehabilitation processes; Practical guidance through health care and community services.
**Emily H. L** [38] **2015**	Canada	5 stroke survivors aged 68 (53–69), Months post discharge 6 (0.5–9)	Framework analysis	In-depth interviews	To determine the experiences and needs of Chinese stroke survivors and family caregivers as they return to community living	Information and training needs during the rehabilitation and return to community living phases; care environments adopted their original culture (communication with professionals, traditional Chinese healing methods, Chinese diet)
**Tina Taule** [49] **2015**	Norway	8 participants (45–80 years), discharge from hospital<6 months	Framework analysis	Semi-structured interviews	To explore mild-to-moderate stroke survivors’ experiences	Hope for a life worth living (continued engagement in activities, altered body and emotional reactions); the trauma of a changed body: making sense of emotional Reactions (questions about uncertainties of life and death, e.g., being afraid of having another stroke); the challenge of cultivating mutual confidence
**Silvio Simeone** [50] **2014**	Italy	15 patients aged 70 (34– 85 years); three months after being discharged home	Phenomenological methodology	Survey with open-ended question	To describe the experience of stroke survivors ©	Need stay at safety; The house suit to my situations, physical and cognitive recovery;
**Randi Martinsen** [51] **2015**	Norway	16 patients aged 48 (21–67 years) (8 patients had lived with the stroke for approximately 1.5 years. The other 8 participants had experienced stroke from 2 to 10 years)	Hermeneutic-phenomenological analysis	In-depth qualitative interviews	To explore young and midlife stroke survivors’ experiences with the health services and to identify long-term follow-up needs	To gain access to follow-up health services tailoring to the individual (e.g., sufficient information, timely and management intervention, instead of examination only); follow-up system had to be flexible to suit their family life situation and disease severity.
**Jennifer H. White** [42] **2014**	Australia	8 participants with UI aged 69 to 88 years; in 4 years since first stroke.	Thematic analysis	Semi-structured interviews	To determine the experiences of community-dwelling stroke survivors living with UI/PSU	Insufficient advice and information from the health system to manage the unpredictable and persisting nature of UI/PSUI symptoms
**Jennifer White** [52] **2014**	Australia	14 responders aged 73.43 (58–89) with an average of three years post-stroke	Grounded theory	Semi-structured interviews	To explore the physical and psycho-social functioning status of stroke survivors beyond 12 months post-stroke and to qualitatively explore the longer-term experiences of psychological morbidity and service access needs	Rely on other people to assist in maintaining involvement in valued pre-stroke life roles; management, monitoring, and interventions about symptoms; to access needed services when services and information were limited
**Marianne E Klinke** [41] **2014**	Iceland	7 stroke survivors aged 53 (34–64) with eating difficulties; mean three years since the stroke	Phenomenological	In-depth interviews	To explore and describe the experience of eating and eating-related difficulties in stroke survivors living at home	Individualized long-term support from family; eating normally and safely not only at home; to maintain a good outward façade and to eat in a socially acceptable way
**Barbara J. Lutz** [33] **2011**	America	19 recovering stroke patients, no detail period post-stroke	Grounded theory	Group and individual interview	To explore the needs of stroke patients and their family caregivers	Keep safety with the help of family (e.g., falling, picking up something); need for assistance with ADLs and IADLs; the support of family and/or friends
**L. Salisbury** [34] **2010**	America	13 patients aged 64.37 (43–75 years), mean time since stroke was 4.63 years;	Interpretative phenomenological analysis	Semi-structured interviews	To explore stroke patients and cares’ experiences of the healthcare system	Positive community rehabilitation services; required encouragement to access leisure facilities and activity; enough follow up and backup car; continuous services and intervention
**Jan Pringle** [35] **2010**	England	4 patients with aphasia, over 18 years of age, one month after discharge	Phenomenological approach	In-depth interviews and self-report diaries	To explore personal experiences of coming home	Talking with the therapists; having a reliance on others to fill limited ability; follow-up is still expected
**J Ö RGEN MEDIN** [53] **2010**	Sweden	13 participants 6 months after last stroke	Grounded theory	Semi-structured interviews	To explore the experience and management of eating situations among persons affected by stroke	No embarrassing and undesirable due to needing help about eating; mastering of eating situations in front of unfamiliar people
**Jacki Liddle** [40] **2009**	Australia	24 participants who had ceased driving, mean of 5.5 years following a stroke, aged 67 (50–83 years)	Phenomenological approach	Semi-structured interviews	To explore the needs and experiences of people who cease driving following a stroke	Information sharing was less effective in early recovery; a detailed explanation of stopping driving not just according to the renewal form; a lack of follow-up regarding the driving cessation process by health professionals; a lack of information given regarding alternative transport options and safely return to driving; the need for different types of transport and other lifestyle changes
**Claude Vincent** [18] **2007**	Canada	17 persons aged from 65 to 85 years with over two years history	Framework analysis	Focus group	To explore partially met and unmet rehabilitation needs of older adults	Receiving home care assistance from the public health care system; motor activities
**Rose Wiles** [36] **1998**	England	8 patients, no detail of age, 2–12 months post-discharge	Grounded theory	Semi-structured interview	To identify a range of information needs that patients and carers may have at three different phases post-stroke	Need for practical information relating to coping with day-to-day activities both in and outside the home; information regarding house adaptations; equipment and the services and resources available in the community.
**Jacqueline McLean** [37] **1991**	England	20 patients, aged from 78 to 69. No detail period post-stroke	Content analysis	semi-structured interviews	To identify service needs of stroke survivors and their informal carers	Affective needs (emotionalism and communication); out-patient follow-up

NIHSS = National Institute Health Stroke Scale; mRS = modified Rankin Scale; UI = urinary incontinence; RTW = Return to Work; ADL = Ability Daily Life.

**Table 2:** Quality Assessment of Involved studies (N = 24).

Study	Q1	Q2	Q3	Q4	Q5	Q6	Q7	Q8	Q9	Q10
Valérie Poulin et al. [44]	Y	Y	Y	Y	Y	Y	UN	Y	Y	Y
Monique R. Pappadis et al. [46]	Y	Y	Y	Y	Y	Y	UN	Y	Y	Y
Emma K. Kj Ö rk et al. [39]	Y	Y	Y	Y	Y	Y	UN	Y	Y	Y
Gunvor GARD et al. [43]	Y	Y	Y	Y	Y	Y	Y	Y	Y	Y
Jamuna Rani Appalasamy et al. [19]	Y	Y	Y	Y	Y	Y	Y	Y	Y	Y
Abrahamson, Vanessa et al. [15]	Y	Y	Y	Y	Y	Y	N	Y	Y	Y
Jessica Shipley et al. [47]	Y	Y	Y	Y	Y	Y	UN	Y	Y	Y
Shannon R.L et al. [48]	Y	Y	Y	Y	Y	Y	N	Y	Y	Y
Nadia Davoody et al. [45]	Y	Y	Y	Y	Y	Y	Y	Y	Y	Y
Emily H. L et al. [38]	Y	Y	Y	Y	Y	Y	Y	Y	Y	Y
Tina Taule et al. [49]	Y	Y	Y	Y	Y	Y	Y	Y	Y	Y
Silvio Simeone et al. [50]	Y	Y	Y	Y	Y	Y	Y	Y	Y	Y
Randi Martinsen et al. [51]	Y	Y	Y	Y	Y	Y	Y	Y	Y	Y
Jennifer H. White et al. [42]	Y	Y	Y	Y	Y	Y	Y	Y	Y	Y
Jennifer White et al. [52]	Y	Y	Y	Y	Y	Y	UN	Y	Y	Y
Marianne E Klinke et al. [41]	Y	Y	Y	Y	Y	Y	UN	Y	Y	Y
Lutz, B J et al. [33]	Y	Y	Y	Y	Y	Y	UN	Y	Y	Y
L. Salisbury et al. [34]	Y	Y	Y	Y	Y	UN	Y	Y	Y	Y
Jan Pringle et al. [35]	Y	Y	Y	Y	Y	Y	Y	Y	Y	Y
J Ö RGEN MEDIN et al. [53]	Y	Y	Y	Y	Y	N	Y	Y	Y	Y
Jacki Liddle et al. [40]	Y	Y	Y	Y	Y	Y	Y	Y	Y	Y
Claude Vincent et al. [18]	Y	Y	Y	Y	Y	Y	Y	N	Y	Y
Rose Wiles et al. [36]	Y	Y	Y	Y	Y	UN	UN	Y	Y	Y
Jacqueline McLean et al. [37]	Y	Y	UN	Y	N	N	UN	N	Y	Y

UN = UNCLEAR; UA = Not applicable; Q1. Is there congruity between the stated philosophical perspective and the research methodology? Q2. Is there congruity between the research methodology and the research question or objectives? Q3. Is there congruity between the research methodology and the methods used to collect data? Q4. Is there congruity between the research methodology and the representation and analysis of data? Q5. Is there congruity between the research methodology and the interpretation of results? Q6. Is there a statement locating the researcher culturally or theoretically? Q7. Are the influences of the researcher on the research and vice-versa, addressed? Q8. Are participants, and their voices, adequately represented? Q9. Is the research ethical according to current criteria or, for recent studies, and is there evidence of ethical approval by an appropriate body? Q10. Do the conclusions drawn in the research report flow from the analysis or interpretation of the data?

## Data Availability

All data relevant to the study are included in the article or uploaded as supplementary information.

## Supplementary Materials

The following are available online at https://www.mdpi.com/1660-4601/18/4/2140/s1, Table S1: JBI Critical Appraisal Checklist for Systematic Reviews and Research Syntheses.
