# Supplementary material for: The Unmet Needs of Community-Dwelling Stroke Survivors: A Systematic Review of Qualitative Studies
Source: Int J Environ Res Public Health. 2021 Feb 22;18(4):2140. doi: 10.3390/ijerph18042140 (PMC7926407; doi:10.3390/ijerph18042140)
Supplement: Supplementary file 1 [file ijerph-18-02140-s001.pdf]

Table S1. JBI Critical Appraisal Checklist for Systematic Reviews and Research Syntheses

	Yes	No	Unclear	Not applicable
1. Is the review question clearly and explicitly stated?	√			
2. Were the inclusion criteria appropriate for the review question?	√			
3. Was the search strategy appropriate?	√			
4. Were the sources and resources used to search for studies adequate?	√			
5. Were the criteria for appraising studies appropriate?	√			
6. Was critical appraisal conducted by two or more reviewers independently?	√			
7. Were there methods to minimize errors in data extraction?	√			
8. Were the methods used to combine studies appropriate?	√			
9. Was the likelihood of publication bias assessed?	√			
10. Were recommendations for policy and/or practice supported by the reported data?	√			
11. Were the specific directives for new research appropriate?	√			

Overall appraisal: Include ☑ Exclude ☐ Seek further info ☐
